# Characteristics of relapsed and refractory paediatric Hodgkin lymphoma; a 10-year retrospective study of an LMIC

**DOI:** 10.3332/ecancer.2024.1729

**Published:** 2024-07-16

**Authors:** Quratulain Riaz, Rabel Gul, Vashma Junaid, Wasfa Farooq, Naema Khayyam

**Affiliations:** Department of Paediatric Oncology, Indus Hospital & Health Network, Karachi, Sindh 75190, Pakistan; a https://orcid.org/0009-0002-3161-7508; b https://orcid.org/0000-0001-6247-3311

**Keywords:** Hodgkin lymphoma, relapse, refractory disease, outcomes

## Abstract

**Background:**

With conventional standard treatment modalities, children and adolescents with Hodgkin lymphoma (HL) have long-term overall survival rates of over 90%. However, primary refractory disease may occur in 5%–10% of HL patients, while relapse occurs in 5%–10% of patients with early stage disease and up to 30% in an advanced stage. This retrospective study evaluated patient characteristics in cases of HL relapse and refractory and their response to second-line treatment of standalone chemotherapy or in combination with radiotherapy.

**Methodology:**

A retrospective study was conducted by Indus Hospital and Health Network to determine the outcomes of paediatric patients with first and second relapses of HL between 2013 and 2022.

**Results:**

A total of 742 patients were diagnosed with HL at Indus Hospital & Health Network. Of these, 48 (6.5%) patients presented with relapse and 35 (4.7%) with refractory disease after initial chemotherapy. In HL relapse patients, 57% were stage IV at initial diagnosis with the most common pathology being nodular sclerosis constituting 42.9% of patients. The most common age group was 6–10 years, 45.8%. B symptoms were experienced by 25 (52%) patients. A time to relapse of >12 months following diagnosis was seen in 69% and 3–12 months was seen in 31%.

After receiving second-line treatment, complete remission was achieved by 34 (70.8%) patients, partial remission (PR) was seen in 5 (10.4%), disease progression in 5 (10.4%), 3 (6.3%) patients left during treatment and 1 (2.1%) had a treatment-related mortality. Re-radiation in second-line treatment was only required for 2 patients. The second relapse was seen in 11 (28.2%) of 39 complete and PR patients.

**Conclusion:**

Major limitations in the treatment of HL relapse in a low-resource setting are the non-availability of immunotherapy and autologous stem cell transplantation due to extreme financial burden and lack of capacity in facilities. Dedicated efforts are required to provide these facilities free of cost in low–middle income countries (LMICs).

## Introduction

Lymphoma is the most frequently occurring cancer in adolescents, with Hodgkin lymphoma (HL) forming two-thirds of the cases [1]. HL is classified histopathologically into two types: Classical HL (cHL) and Nodular lymphocyte-predominant HL, with cHL accounting for 95% of cases and including the subtypes nodular sclerosing, mixed cellularity, lymphocyte-rich and lymphocyte-depleted HL [2].

With conventional standard treatment modalities, children and adolescents with HL have long-term overall survival (OS) rates of over 80% [3]. However, primary refractory disease (that either does not respond to initial therapy or progresses after an initial partial response) may occur in 5%–10% of HL patients, while relapse (reappearance of disease at sites of prior disease and/or at new sites after the achievement of complete remission (CR)) occurs in 5%–10% of patients with early-stage disease and up to 30% in advanced stage [4].

The goal of salvage therapy for relapsed or refractory HL is to achieve long-term disease control while limiting toxicity and complications of the treatment [5]. Salvage therapy options include additional standard dosage chemotherapy, consolidative radiotherapy, autologous or allogeneic stem cell transplantation (SCT), and more recently, immunotherapy with medicines such as immune checkpoint inhibitors brentuximab vedotin [6]. However, no conclusive evidence suggests that any particular treatment is better than the others [4, 7].

Most of the published literature on the treatment for HL relapse and refractory disease pertains to salvage therapy using SCT. There is not enough reported data to establish the factors that are sufficient in identifying the patients who are at a high risk of relapsing and who need intensification of therapy [8]. Furthermore, the survival rate of HL recurrence in developing nations is unclear [9]. As the Indus Hospital & Health Network (IHHN) receives a substantial number of HL patients we intend to investigate this rate through our research.

The aim of our study was to study the influence of clinical characteristics on the effectiveness of salvage therapy for patients with relapsed HL.

## Methodology

A retrospective study was conducted at IHHN, Karachi to determine outcomes of pediatric patients with HL between 2013 and 2022. Medical records were reviewed of patients under 18 with primary HL who had either relapsed after achieving a first remission or were refractory to treatment. Initial treatment of all patients consisted of 4–8 alternating courses of Adriamycin, Bleomycin, Vincristine, Dacarbazine/Cyclophosphamide, Vincristine, Prednisolone and Dacarbazine (COPDAC) with radiation therapy given only to patients with significant residual disease on computed tomography scan at the end of our treatment regimen. This was an institutional protocol due to excessive toxicity to OEPA [Vincristine sulfate (Oncovin), Etoposide, Prednisone, and Doxorubicin Hydrochloride (Adriamycin)]/COPDAC. Relapse was identified by the presence of new clinical or radiological lesions on surveillance ultrasound or X-ray and proven by lymph node biopsy or bone marrow biopsy. Salvage therapy consisted of either standalone chemotherapy or chemotherapy with radiotherapy. The chemotherapy regimen for second line or salvage therapy included; Etoposide, prednisolone, Ifosfamide, Cisplatin (EPIC) or Gemcitabine/Vinorelbine (GEM/VINO) combination Regimen depending on the availability of the drug and socioeconomic factors of the patient. Low-risk patients were assigned four cycles, intermediate risk assigned six cycles and high risk assigned eight cycles. Interim assessment was done after four cycles of chemotherapy in each patient across the 10-year period. Data variables assessed were gender, pathology type, stage of cancer, interim response, presence of B symptoms in both primary and relapsed HL, time to relapse, mediastinal disease at relapse, chemotherapy regimens, radiotherapy treatment and outcomes at the end of treatment.

The time to first relapse was calculated from the date of the end of primary treatment to relapse. Early relapse was defined as the number of patients who relapsed between 3 and 12 months of CR after primary therapy, whereas late relapse was defined as recurrence after 12 months of achieving CR [4]. Patients who either did not respond to initial therapy or progressed after an initial partial response within 3 months were considered refractory. Response assessment was done by positron emission tomography-computed tomography (PET CT) scan with interim assessment being done after two cycles of second-line chemotherapy. OS was measured from the date of relapse until death. Progression-free survival (PFS) was measured from the first relapse until death or the second relapse.

All analyses were performed using STATA software version 15. The study was approved by the hospital’s Institutional Review Board. Informed consent for treatment was obtained from the parents or legal guardians.

## Results

**Figure 1.**
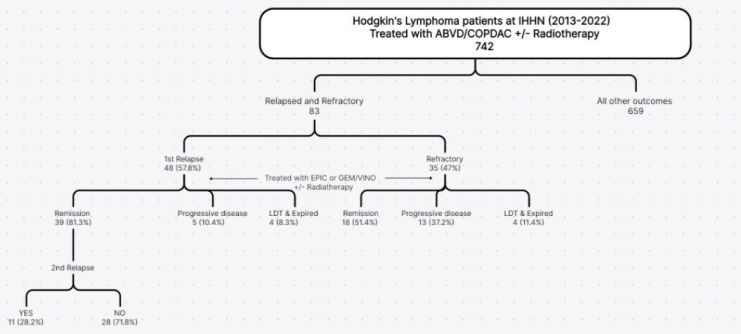
Outline of relapsed and refractory HL pediatric patients.

A total of 83 patients with HL at IHHN from 2013 to 2022 were included in the study, 67 (80.7%) were male and 16 (19.3%) were female. After the first chemotherapy, 48 (57.8%) of these patients had relapsed and 35 (47%) had a refractory illness. A brief outline of the relapsed and refractory cases has been shown in Figure 1.

### Clinical characteristics

In HL relapse patients, 35 (42.2%) were stage IV at initial diagnosis with the most common pathology being nodular sclerosis in 35 (42.2%) patients. B symptoms were found in 29 (34.9%) patients with a first-time relapse and a time to relapse of 3–12 months following diagnosis was seen in 31% and >12 months in 69% of patients, with a median time to relapse of 18 months with an interquartile range of 19.3 months. Age at relapse ranged from 5 to 19 years with a mean of 9.95 ± 3.62 years. Risk factors at initial diagnosis were compared with patients that relapsed or became refractory; Age at relapse (*p* = 0.009), response to treatment on interim assessment (*p* < 0.001), radiation received (*p* < 0.001) and status at end of treatment (*p* < 0.001) all showed highly significant values on comparison between relapse and refractory HL (Table 1). This was also observed in time to relapse, with significant differences in interim assessment (*p* = 0.016), inclusion of radiation (*p* < 0.001) and treatment outcome (*p* < 0.001) among patients with either first or second relapse (Table 2).

### Treatment course

Second-line chemotherapy of either GEM/VINO or EPIC was given. Radiation after chemotherapy in initial treatment was given to 26 (31.3%) patients, out of which 21 patients (80.1%) were refractory and the remaining achieved first remission. Re-radiation in second-line treatment was only required for two patients.

### Outcomes

**Figure 2.**
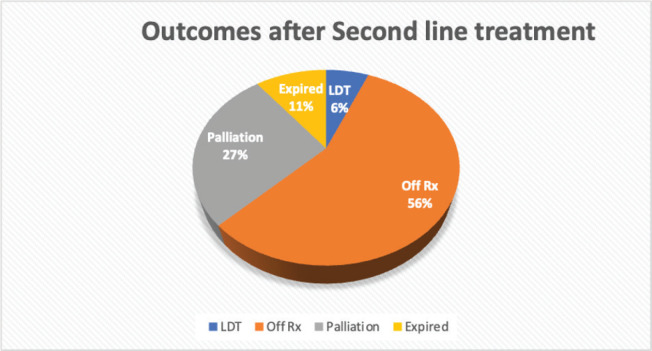
Outcomes of patients after receiving second-line treatment.

Second relapse was seen in 11 (28.2%) of 39 complete and partial remission (PR) patients. The characteristics of patients who had second relapse are further outlined in Table 3. After receiving second-line treatment, CR was achieved by 34 (70.8%) patients, PR or no change was seen in 5 (10.4%), disease progression in 5 (10.4%), 3 (6.3%) patients left during treatment and 1 (2.1%) was treatment-related mortality. Overall treatment outcomes are shown in Figure 2. We compared the outcomes of relapsed patients with second-line chemotherapy GEM/VINO and EPIC treatment; however, no significant association was found with recurrence of relapse (*p*-value of 0.093) nor with the status end of treatment (*p*-value of 0.123).

**Figure 3.**
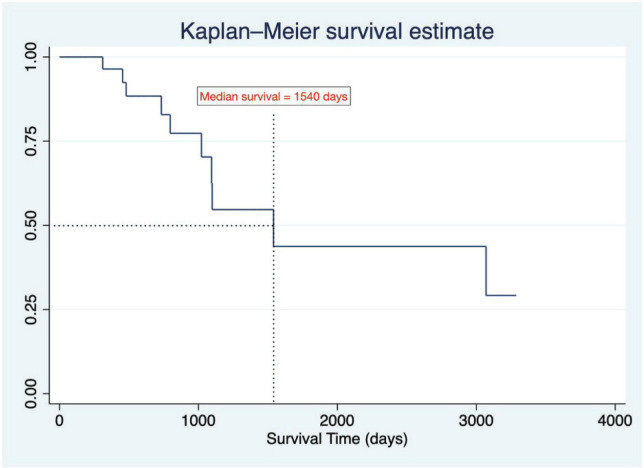
Kaplan-Meier survival estimate.

The average PFS, measured from the first relapse to the second relapse or death, was 10.5 ± 6.75 months. The average OS rate, from the date of relapse to death, was 51.2 ± 28.2 months. A Kaplan-Meier survival graph (Figure 3) was plotted for the event of death. The estimated median survival time was 1,540 days. The probability of a death event in relapse patients at the end of the study period was approximately 0.3125 or 30%.

## Discussion

With the progress of cancer treatments, outcomes of HL have witnessed significant improvements, even with standalone chemotherapy [10]. However, refractory or recurrent HL is still considered to affect about 15% of all patients [11]. According to the findings of this study, 11.1% of individuals from our 10-year cohort experienced relapse or had refractory disease.

In patients presenting with relapse of disease, the most frequently observed age interval was 6–10 years of age, 31 (65%), and the most common stage of disease was stage IV, noted in 23 (48%) cases. Whereas, in refractory patients ages 11–15 years old were the most common (57%) with stage III (43%) being the most frequent. Global data also reports similar findings with stage IV being the most common stage at relapse [12–15]. According to the literature, the median age at relapse varied depending on the socioeconomic condition of the country. A multicenter study done in the United States in 2013 reported a median age at relapse of 17.3 years [12]. Other studies from developed nations also showed higher ages at relapse [16, 17]. Whereas the mean age at relapse ranged from 8 to 10 years in underdeveloped countries [18–20], similar to our findings.

Within our patient cohort, we employed two chemotherapy regimens for relapse patients: EPIC and GEM/VINO. The response rate observed was an impressive 80% which was similar to a response rate of 83% reported by another study that explored the response rates with GEM/VINO as a second-line chemotherapy in pediatric HL [21]. In our study, there was a CR rate of 58% and a PR of 22%. However, 13 (27%) patients exhibited progressive illness despite treatment. In cases of early relapse (within 3–12 months), 11 (65%) achieved CR and 6 (35%) showed PR. On the other hand, in instances of late relapse (after 12 months), 31 (89%) achieved CR, while 4 (11%) experienced PR.

Interestingly, our analysis revealed no significant correlation between EPIC and GEM/VINO treatment outcomes in relation to relapse. Consequently, it cannot be concluded that one treatment is superior to the other, and no specific recommendation can be made. These findings seem consistent with a study done in Shaukat Khanum Memorial Cancer Hospital and Research Centre, Lahore, from September 2009 to December 2013 reported a CR of 68% and PR of 24% with chemotherapy regimens EPIC, GEM/VINO and DHAC (Dexamethasone Cytarabine Carboplatin) [11].

The International Prognostic Score was created to better identify HL patients with an unfavourable prognosis who could benefit from increased therapy [22]. Presently, no such score exists for paediatric HL. A Memorial Sloan-Kettering Cancer Centre study including young adults with relapsed or refractory disease, investigated the effect of two biweekly rounds of Ifosfamide, carboplatin and etoposide. B symptoms, extranodal disease and an early relapse were observed to be negative prognostic factors [23]. This is similar to our findings, where the presence of B symptoms showed an association with staging of disease (*p* = 0.004) and time to relapse of 3–12 months resulted in 47% of patients discontinuing their treatment with a high number of deaths (23%) and 23% patients needing palliative care. Euronet stated time to relapse as the most important prognostic factor [15]. In adult HL patients, three key predictive markers for response to second-line therapy are used: age (> 40 or 50 years), stage of recurrence and type of medication used to treat relapse [24]. However, the use of these key predictors for paediatric cases is still mostly unexplored. By evaluating risk factors, they were able to predict which patients were at a high risk of relapse and needed treatment with more advanced methods. A similar model can be of great use for pediatric oncology in low–middle income countries (LMICs) where there are severe limitations in resources and an overburden of patients in the few existing centers. As shown in this study, significant risk factors that may be included are age (*p* < 0.001) and results of the interim assessment (*p* = 0.016). A study by Ghafoor [25] at Combined Military Hospital Rawalpindi, Pakistan, also explored prognostic factors in pediatric HL. Interestingly they found malnutrition to be a negative factor that was not explored in our study. Their study also reported the advanced stage of disease being a negative prognostic factor which was in line with our study [25]. Further studies should be done to investigate further clinical correlations with prognostic significance in childhood cancer.

According to our study's findings, the outcomes for patients varied considerably. Among relapsed patients, 56% successfully discontinued therapy, while only 25% of refractory cases achieved the same outcome. Unfortunately, the mortality rate for refractory individuals was 25%, whereas 10% for those who relapsed. Furthermore, 37% of refractory patients needed palliative care, while 14% left during treatment. Future studies must be done to track outcomes and compare existing treatment regimes with newer drug options. The DAL/GPOH-HD Study Group incorporated SCT for HL patients with unfavourable prognoses, leading to improved OS [26]. Numerous other studies also state autologous stem cell transplantation (ASCT) as a superior treatment modality to traditional salvage chemotherapy [13, 27, 28]. However, in LMICs, access to ASCT is often not available or affordable for most patients. Newer treatments such as brentuximab vedotin and immune checkpoint blockers such as Nivolumab and Pembrolizumab have also shown high CR rates and PFS values [29–31].

Although our study provides valuable insights into observed outcomes and risk factors for relapsed and refractory HL in an LMIC, there are noticeable challenges in anatomising definitive correlations and prognostic factors which are imperative for improving patient outcomes and the homogeneity of treatment protocols. Our findings regarding treatment outcomes underscore the challenges in managing refractory cases, with higher mortality rates and a greater need for palliative care compared to relapsed patients. The potential benefits of incorporating SCT and newer treatments like Nivolumab and Brentuximab Vedotin need to be studied further along with their limited accessibility in LMICs.

## Limitations

A significant limitation of our study is the 10-year duration due to which a substantial number of patients were lost to follow up or had incomplete data available. As a result of this, the rate of relapsed or refractory HL in our study is understated, particularly noted in the low number of second-relapse patients.

While the treatment of choice varies depending on several factors in HL relapse or refractory disease, due to resource and logistical limitations, the preferred regimens for specific cases are not always available due to which alternatives to first-line chemotherapy drugs are used instead. This can lead to significant variations in individual outcomes as well as discrepancies in overall comparisons amongst the different treatment modalities, particularly between GEM/VINO and EPIC chemotherapy.

## Conclusion

As this study sheds light on the unique challenges faced by paediatric HL patients in LMICs, it advocates for continued research to track outcomes, compare treatment modalities and explore the feasibility of newer drug options. Ultimately, a comprehensive understanding of risk factors and treatment strategies is crucial for improving standards of care and ensuring the most effective and accessible interventions for paediatric HL in diverse healthcare settings.

## List of abbreviations

ASCT, Autologous stem cell transplantation; cHL, Classical Hodgkin lymphoma; EPIC, Etoposide, prednisolone, Ifosfamide, Cisplatin; GEM/VINO, Gemcitabine/Vinorelbine; IHHN, Indus Hospital and Health Network; LMIC, Low-middle income country.

## Conflicts of interest

The authors of this study affirm that there are no conflicts of interest to report pertaining to this research, including but not limited to any financial, personal or professional interests that could pose a conflict with the objectivity, integrity or impartiality of this research project.

## Funding

There is no funding or financial support for this study to be declared.

## Author contributions

The authors confirm contribution to the paper as follows: study conception and design: Naema Khayyam, Quratulain Riaz, Rabel Gul, Vashma Junaid, Wasfa Farooq; data collection: Vashma Junaid, Rabel Gul, Wasfa Farooq; analysis and interpretation of results: Wasfa Farooq, Vashma Junaid, Rabel Gul, Naema Khayyam, Quratulain Riaz; draft manuscript preparation: Rabel Gul, Vashma Junaid, Wasfa Farooq, Quratulain Riaz, Naema Khayyam. All authors reviewed the results and approved the final version of the manuscript.

All authors mentioned in our submission have contributed to the manuscript in significant ways. Furthermore, we have individually and collectively reviewed and agreed upon the final manuscript content. We have no conflicts of interest to disclose.

## Figures and Tables

**Table 1.** Patient characteristics at initial diagnosis for relapsed and refractory HL.

	*N* (%)		*p* value
	Relapse	Refractory	
Gender			0.204
Male	41 (86)	26 (74)	
Female	7 (15)	9 (26)	
Total	48 (100)	35 (100)	
Age intervals (years)			0.009
0–5	2 (4)	1 (3)	
6–10	31 (65)	10 (29)	
11–15	12 (25)	20 (57)	
>15	3 (6)	4 (11)	
Total	48 (100)	35 (100)	
Stage			0.597
I	2 (4)	3 (9)	
II	6 (13)	5 (14)	
III	17 (35)	15 (43)	
IV	23 (48)	12 (34)	
Total	48 (100)	35 (100)	
Pathology			0.694
Nodular sclerosis	20 (42)	14 (40)	
HL mixed	10 (21)	10 (29)	
HL unspecified	18 (38)	11 (31)	
Total	48 (100)	35 (100)	
B symptoms			0.824
Yes	34 (71)	24 (69)	
No	14 (29)	11 (31)	
Total	48 (100)	35 (100)	
Interim assessment			<0.001
Adequate response	37 (77)	1 (3)	
Inadequate response	11 (23)	31 (89)	
Progressive disease	0 (0)	3 (9)	
Total	48 (100)	35 (100)	
Radiation received			<0.001
Yes	5 (11)	21 (60)	
No	43 (90)	14 (40)	
Total	48 (100)	35 (100)	
Status end of treatment			<0.001
CR	42 (88)	6 (19)	
PR/ No change	6 (13)	12 (39)	
Progressive	0 (0)	13 (42)	
Total	48 (100)	31 (100)	
Final outcome (define in discussion			0.039
Alive off treatment	27 (56)	9 (25)	
Expired	5 (10)	8 (25)	
LDT	3 (6)	5 (14)	
Palliative	13 (27)	13 (37)	
Total	48 (100)	35 (100)	

**Table 2.** Comparison of time to recurrence of disease with patient characteristics.

	Time to recurrence (months)				*p* value
	< 3 (refractory)	3-12 (early relapse)	>12 (late relapse)	Total	
Gender					0.566
Male	7 (100)	15 (88)	30 (86)	52	
Female	0 (0)	2 (12)	5 (14)	7	
Total	7 (100)	17 (100)	35 (100)	59	
Stage					0.495
I	1 (14)	1 (6)	1 (3)	3	
II	0 (0)	3 (18)	4 (11)	7	
III	2 (29)	8 (47)	11 (31)	21	
IV	4 (57)	5 (29)	19 (54)	28	
Total	7 (100)	17 (100)	35 (100)	59	
Interim assessment					0.016
Adequate response	1 (14)	10 (59)	27 (77)	38	
Inadequate response	5 (71)	7 (41)	7 (20)	19	
Progressive disease	1 (14)	0 (0)	1 (3)	2	
Total	7 (100)	17 (100)	35 (100)	59	
Radiation					<0.001
Yes	6 (86)	3 (17)	5 (14)	14	
No	1 (14)	14 (82)	30 (86)	45	
Total	7 (100)	17 (100)	35 (100)	59	
Status end of treatment					<0.001
CR	1 (14)	11 (65)	31 (89)	43	
PR/no change	6 (86)	6 (35)	4 (11)	16	
Total	7 (100)	17 (100)	35 (100)	59	
Final outcome					0.411
Alive off treatment	2 (28)	8 (47)	19 (54)	29	
Expired	3 (42)	4 (23)	3 (8)	10	
LDT	0 (0)	1 (5)	2 (5)	3	
Palliative	2 (28)	4 (23)	11 (31)	17	
Total	7 (100)	17 (100)	35 (100)	59	

**Table 3.** Patient characteristics for second relapse patients.

	Second relapse		*p* value
	Yes	No	
Gender			0.174
Male	8 (73)	33 (89)	
Female	3 (27)	4 (11)	
Total	11 (100)	37 (100)	
Pathology			0.950
HL nodular sclerosis	5 (45)	15 (41)	
HL mixed	2 (18)	8 (22)	
HL unspecified	4 (36)	14 (38)	
Total	11 (100)	37 (100)	
Stage			0.622
I	1 (9)	1 (3)	
II	1 (9)	5 (14)	
III	5 (45)	12 (32)	
IV	4 (36)	19 (51)	
Total	11 (100)	37 (100)	
Status end of treatment			0.697
CR	10 (90)	32 (86)	
PR	1 (9)	5 (13)	
Total	11 (100)	37 (100)	
Final outcome			<0.001
Alive off treatment	0 (0)	27 (73)	
Expired	1 (9)	4 (11)	
Palliative	10 (91)	6 (16)	
Total	11 (100)	37 (100)	
